# Effects of a training program for occupational health professionals on the cognitions and perceptions of workers: a randomized controlled trial

**DOI:** 10.1007/s00420-021-01823-7

**Published:** 2022-01-13

**Authors:** Mariska de Wit, Bedra Horreh, Carel T. J. Hulshof, Haije Wind, Angela G. E. M. de Boer

**Affiliations:** Amsterdam UMC, Department of Public and Occupational Health, University of Amsterdam, Coronel Institute of Occupational Health, Amsterdam Public Health Research Institute, PO Box 22700, 1100 DE Amsterdam, The Netherlands

**Keywords:** Occupational health, Occupational physicians, Insurance physicians, Cognition, Perception, Training program

## Abstract

**Purpose:**

To evaluate the effects of a training program for occupational health professionals (OHPs) on their ability to identify the cognitions and perceptions of workers with a chronic disease that may hinder work participation, and on their ability to recommend evidence-based interventions aimed at the identified cognitions and perceptions.

**Methods:**

A randomized controlled trial was conducted in which OHPs were randomly assigned to a training program on the cognitions and perceptions of workers with a chronic disease (*n* = 29) or to a control group that did not receive training (*n* = 30). Participants received home assignments in which they had to identify the cognitions and perceptions of workers in video vignettes and had to indicate which interventions they would recommend to foster work participation. A generalized linear model repeated measures ANOVA was conducted to study the effects of the training program.

**Results:**

The results of the analyses showed an increase in the ability to identify the cognitions and perceptions of workers of OHPs who received the training compared to the control group (*p* < 0.001). The results also showed an increased ability to recommend evidence-based interventions aimed at these cognitions and perceptions (*p* < 0.001) as a result of participation in the training.

**Conclusion:**

The training program helps OHPs to identify cognitions and perceptions and to recommend evidence-based interventions. This can support them in their activities to increase the work participation of workers with a chronic disease.

**Supplementary Information:**

The online version contains supplementary material available at 10.1007/s00420-021-01823-7.

## Introduction

It is expected that the prevalence of chronic diseases, here defined as diseases with a long duration and slow progression, in adults of working age will increase because of the aging world population and an increase in the state pension age in different countries (De Vroome et al. 2015; World Health Organisation 2021). However, having a chronic disease has a negative effect on work participation (De Vroome et al. 2015; Scharn et al. 2019). Nonetheless, people with a chronic disease greatly value their work, for example, for providing them with an income, social contacts, and the feeling that they contribute to society (Vooijs et al. 2018). Sickness absence due to chronic diseases can have a large financial burden (De Vroome et al. 2015). Therefore, the work participation of people with a chronic disease, should be supported.

To support work participation, occupational health professionals (OHPs)—namely health professionals who make decisions about work participation or workers receiving benefits—should focus on factors that influence work participation. Workers’ cognitions and perceptions are factors that can influence their work participation (De Wit et al. 2018; Besen et al. 2015). For example, fear-avoidance beliefs have a negative effect on return to work (RTW) after sick leave, while positive expectations regarding RTW have a positive effect on work participation (Trinderup et al. 2018; Opsahl et al. 2016). OHPs should take these factors into account in order to stimulate work participation in workers with a chronic disease.

However, taking cognitions and perceptions into account during consultations can be a challenge. Various factors can make it difficult to obtain information on cognitions and perceptions from workers. Factors such as a lack of trust in the OHP or a lack of empathy by the OHP may hinder disclosure by workers, which may limit the information that OHPs obtain concerning important cognitions and perceptions (Dalma et al. 2020; Kelak et al. 2018; De Wit et al. 2019a). If OHPs obtain information about the cognitions and perceptions of workers successfully, another challenge for them is to know what to do when these factors limit work participation.

To overcome these difficulties, a training program was developed for OHPs on how to identify cognitions and perceptions during consultations and on recommending interventions aimed at these factors. The content of the training program is evidence-based, but we do not know whether this training has an effect on the ability of OHPs to identify cognitions and perceptions that limit work participation and to recommend evidence-based interventions to change them. Therefore, in this study we evaluate the effectiveness of the training in a randomized controlled trial.

The research questions are: does the newly developed training program for OHPs have an effect on the ability to identify the cognitions and perceptions of workers? And does the training have an effect on the ability of OHPs to recommend evidence-based interventions toward workers aimed at the cognitions and perceptions of workers?

## Methods

### Study design

In this randomized waiting-list controlled trial, participants in the intervention group participated in the training program in October 2019. Participants in the control group participated in November 2019 after completing the post-test. During this study period, no restrictions were imposed on participants with regard to following any other training. The Medical Ethics Review Committee of the Academic Medical Center (AMC), University of Amsterdam, confirmed that the Medical Research Involving Human Subjects Act (WMO) did not apply to this study and the official approval of this committee was therefore not required (W 19__174 # 19.213). The Consolidated Standards of Reporting Trials (CONSORT) Statement was used for reporting (Schulz et al. 2010).

### Participants

OHPs were eligible if they were occupational physicians (OPs), OPs in training, insurance physicians (IPs), or IPs in training; these are the main OHPs in the Netherlands. The role of OPs is to prevent occupational and work-related diseases, promote health, prevent sick leave, or promote RTW after sick leave by, for example, recommending interventions that can increase work participation. IPs evaluate the functional abilities and disabilities of workers that can influence whether they receive a work disability benefit and provide recommendations for interventions to promote RTW. Although the roles of OPs and IPs differ, they perceive the same cognitions and perceptions as important for work participation and use the same methods to obtain information about these factors (De Wit et al. 2019b). Therefore, OPs and IPs received the same training.

The participants in this convenience sample were recruited from professional associations and educational institutions in the Netherlands. In July, August, or September 2019, OHPs who were members of the Netherlands Society of Occupational Medicine (NVAB) or the Dutch Association for Insurance Medicine (NVVG) or were in training at the School for Public and Occupational Health Professionals (SGBO) or the Netherlands School of Public and Occupational Health (NSPOH) received an email inviting them to participate. The email provided information about the content, duration, and location of the training, information about the home assignments, and the researchers’ contact details. Members of the Dutch Association of Medical Advisers in Private Insurance (GAV) could access the same information on their organization’s website.

OHPs who were interested in participating had to email the researchers before October 1, 2019. They subsequently received an email containing further information about the training and when it would be held. After signing the informed consent form, which was sent by regular mail, participants were assigned to the control or the intervention group.

### Randomization

The OHPs were randomized by one researcher (MdW) using a randomized block design. A random number generator (www.randomizer.org) was used to assign the participants to one of four training sessions in October 2019 (intervention group) or one of four training sessions in November 2019 (control group), with an allocation ratio of 1:1. We assigned OPs and IPs to their condition separately, to get an equal distribution of OPs and IPs in the control and intervention group. Because the registration for participation was spread over several months (July–September), participants were randomized in different phases, so not all participants had to wait until October to learn the date of their training. Participants were not informed about whether they were in the control group or intervention group until the study was completed.

### Training program

The evidence-based training program has a duration of 4.5 h, including breaks of in total 30 min, and consists of four parts (Table 1). First, participants learn about ten cognitions and perceptions that are important for work participation of workers with a chronic disease: recovery and RTW expectations, self-efficacy, feelings of control, perceived health, fear-avoidance beliefs, perceived work-relatedness, coping strategies, catastrophizing, motivation and optimism/pessimism (De Wit et al. 2018). They furthermore discuss the importance of these factors with each other. Second, they learn how to obtain information about cognitions and perceptions. Participants practice with identifying cognitions and perceptions in written cases. Furthermore, they are asked to produce questions which they can ask to workers to obtain information regarding these factors. Participants also receive a conversation tool including an overview of the cognitions and perceptions and indicators for limiting or promoting cognitions and perceptions. In the third part, participants learn about factors that can influence the course of the conversation concerning these factors. The information for the second and third parts of the training program was derived from a questionnaire study among OHPs and a focus group study among workers (De Wit et al. 2019a, b). In the final part, participants learn how they can intervene to mitigate limiting cognitions and perceptions. Participants discuss how they have been dealing with workers with limiting cognitions and perceptions. Besides, they learn about interventions from a scoping review and interventions described in guidelines for OHPs (De Wit et al. 2020). For example, they learn about cognitive functional therapy (Vibe Fersum et al. 2013) and a cognitive behavioral group intervention on work anxiety (Muschalla et al. 2016). These interventions can change such cognitions as fear-avoidance beliefs and the perceived work-relatedness of the health problem, and may stimulate work participation. In total, eight training sessions were held in the Amsterdam UMC, the Netherlands.

**Table 1:** Overview of training program on cognitions and perceptions

Four-part evidence-based training program for occupational health professionals		
Part 1	Learning about ten cognitions and perceptions associated with work participation	
	1. Recovery and return to work expectations 2. Self-efficacy 3. Feelings of control 4. Perceived health 5. Fear-avoidance beliefs	6. Perceived work-relatedness 7. Coping strategies 8. Catastrophizing 9. Motivation 10. Optimism/pessimism
Part 2	Learning how information regarding cognitions and perceptions can be obtained	
Part 3	Learning which factors can influence the course of the conversation concerning cognitions and perceptions	
Part 4	Learning how cognitions and perceptions can be changed in order to improve work participation	

### Outcome measures

#### Home assignments

The effects of the training were measured by means of two home assignments. All participants received the first assignment (pre-test: T0) and a questionnaire on demographic variables (name, date of birth, gender, function, years of experience) at baseline. Two weeks after following the training, the OHPs in the intervention group received the second home assignment (post-test: T1). The OHPs in the control group received the same assignment within the same period of time. The assignments were sent by the research assistant, and participants had two weeks to complete and return them.

The home assignments consisted of two questions about four video vignettes of consultations between an OP and a client in which several cognitions and perceptions were incorporated: (A) What cognition(s) or perception(s) of the client do you identify in the video that can influence the client’s work participation? (Mention at least one and a maximum of four); and (B) What intervention(s) would you recommend when this/these cognition(s) or perception(s) limit(s) the client’s work participation, in order to support the work participation? (Mention a maximum of two options per cognition or perception). The videos in the first assignment were different from those in the second assignment, but the content and difficulty were comparable. The OHPs watched the video vignettes in the home assignments for the first time and did not practice with any video vignettes during the training. Therefore, the OHPs were obligated to apply the learned knowledge and skills in a new situation which resembled a real-life consultation.

In exercises A of the four videos (1A, 2A, 3A, and 4A) of the home assignments, the OHPs could score points by identifying the right cognitions and perceptions in the videos. In exercises B of the four videos (1B, 2B, 3B, and 4B), the OHPs could score points by recommending evidence-based interventions aimed at the cognitions and perceptions of the client in the videos. Points were assigned when interventions or components of interventions were mentioned that were aimed at limiting cognitions and perceptions and may increase work participation according to results from a scoping review and guidelines for OHPs (De Wit et al. 2020).

All home assignments were independently checked by two researchers, who received the assignments without demographic data and were blind to the participants’ condition. The final scores on exercises A (0–26 points) and B (0–50 points) of the first assignment and on exercises A (0–24 points) and B (0–44 points) of the second assignment were calculated by taking the mean scores given by the two researchers. The final scores were discussed if they differed by more than three points between the researchers, until consensus on the scoring was reached. Because the total number of achievable points differed between the first and the second assignment, the points were converted into final scores ranging from 0 to 100 by dividing the points by the maximum achievable points and multiplying that figure by 100. A high total score on exercise A indicates a high level of ability to identify cognitions and perceptions, while a high total score on exercise B indicates a high level of ability to recommend evidence-based interventions.

#### Video vignettes

The eight vignettes were videos of simulated consultations between OPs and clients, developed following guidelines suggested by Hillen et al. (2013). In the video vignettes, clients talked about their physical and/or mental health problems, which were cancer, pain in arms and shoulders, pain in neck and back, hernia, cardiovascular disease, stress related complaints and depression. During the talks, various cognitions and perceptions emerged. For example, clients in the video talked about being afraid to RTW because their pain would possibly increase, indicating fear-avoidance beliefs. The video vignettes were based on audio recordings of consultations between an OP and nine clients, all of whom had signed informed consent forms to record their consultations. The scripts were written and discussed by all researchers, of whom one is an OP and one is an IP. Before filming, the scripts were rehearsed with the actors in order to make necessary changes to ensure that the scripts were as realistic as possible.

The video vignettes were recorded in a simulated consulting room by a professional audiovisual production agency. The clients were played by a female and male actor with experience in playing clients. Because the role of the OP in the videos was limited, the OP was played not by a professional actor, but by two researchers from the Amsterdam UMC, who both had experience with conducting consultations as an occupational therapist and a sociotherapist. The duration of the shortest video was 2 min and 39 s, that of the longest 3 min and 3 s. To pilot test the assignments, the video vignettes and assignments were sent to an IP trainer who judged them as appropriate for the assessment of the learned skills in the training.

### Data analysis

Statistical analyses were performed using SPSS statistics 26.0. Characteristics of the participants were described using descriptive statistics. Participants were only included in the analyses if they had completed both the pre-test and post-test. A *t*-test was used to analyze group baseline differences in continuous variables and a chi-square test was used for categorical variables. A generalized linear model (GLM) repeated measures ANOVA was conducted to evaluate the effects of the treatment group (intervention vs. control) and time (pre-test vs. post-test) and time by treatment interaction. Analyses were conducted according to the intention-to-treat (ITT) principle; that is, all participants were analyzed according to the condition in which they were assigned at the beginning of the study. After the ITT analyses, per-protocol (PP) analyses were conducted to test whether the effects of the training were different if we only analyzed the participants who had followed the training program and completed the home assignments in accordance with their condition. The tests were considered significant when *p* < 0.05.

## Results

### Participant characteristics

In total, 62 OHPs were randomly assigned to the control or the intervention group. Fifty-nine physicians completed the pre-test and the post-test; of these physicians, 29 were allocated to the intervention group. Of the 59 participants, three did not adhere to the study protocol. One participant in the intervention group did not participate in the training. Another participant, who was assigned to the control group and should have followed the training in November, followed the training in October, just like the intervention group. Therefore, these two participants were excluded from the PP analyses. One participant in the control group did not participate in the training, but because this had no effect on the scores on both of his home assignments, he was not excluded from the PP analyses. A flowchart of the study is presented in Online Resource 1.

The demographic variables of all participants who completed the pre-test and the post-test are presented in Table 2 by their original assigned groups. There were no significant differences on these variables between the intervention and control group at baseline.

**Table 2:** Demographic variables at baseline

	Total (*N* = 59)		Intervention group (*N* = 29)		Control group (*N* = 30)		*p* value
	*N*(%)	*M* (SD)	*N*(%)	*M* (SD)	*N*(%)	*M* (SD)	
Age		50.8 (11.8)		49.5 (11.5)		52.2 (12.3)	0.385
Gender							
Male	32 (54.2)		13 (44.8)		19 (63.3)		0.154
Female	27 (45.8)		16 (55.2)		11 (36.7)		
Function							
OP	25 (42.4)		12 (41.4)		13 (43.3)		0.840
OP in training	6 (10.2)		4 (13.8)		2 (6.7)		
IP	17 (28.8)		8 (27.6)		9 (30.0)		
IP in training	11 (18.6)		5 (17.2)		6 (20.0)		
Years of work experience		16.9 (12.0)		15.8 (11.4)		18.1 (12.6)	0.474

*M* mean, *SD* standard deviation

### Effects of the training program

#### Effect on the ability to identify cognitions and perceptions (exercise A)

The mean score of the intervention group on exercise A increased from 31.7 (9.2) at pre-test to 55.5 (17.1) at post-test (Table 3). The mean score of the control group on exercise A increased from 29.7 (8.2) to 35.0 (9.6). Results of the GLM repeated measures ANOVA showed a significant effect for Time (*F*(1,57) = 69.2, *p* = 0.000) in ITT analyses on the scores on exercise A. There was also a significant effect for Group (*F*(1,57) = 20.9, *p* = 0.000). There was a significant interaction effect of Time × Group (*F*(1,57) = 28.1, *p* = 0.000). This indicates a positive effect of the training on scores on exercise A. PP analyses also indicated two significant main effects for time and condition and a significant interaction effect.

**Table 3:** GLM repeated measures ANOVA on final scores exercise A and exercise B (ITT analyses)

	T0	T1	*F*	*p* value
	*M *(SD)	*M *(SD)		
Exercise A				
Intervention group	31.7 (9.2)	55.5 (17.1)	T: *F*(1,57) = 69.2	0.000
Control group	29.7 (8.2)	35.0 (9.6)	G: *F*(1,57) = 20.9	0.000
			T × G: *F*(1,57) = 28.1	0.000
Exercise B				
Intervention group	13.9 (4.7)	27.2 (20.2)	T: *F*(1,57) = 5.9	0.018
Control group	13.8 (6.9)	10.0 (8.5)	G: *F*(1,57) = 13.8	0.000
			T × G: *F*(1,57) = 19.0	0.000

*M* mean, *SD* standard deviation, *T* time effect, *G* group effect, *T* × *G* time by group interaction effect

#### Effect on the ability to recommend evidence-based interventions (exercise B)

The mean score of the intervention group on exercise B increased from 13.9 (4.7) at pre-test to 27.2 (20.2) at post-test (Table 3). The mean score of the control group on exercise B was 13.8 (6.9) at pre-test and 10.0 (8.5) at post-test. Results of the GLM repeated measures ANOVA showed a significant effect for Time (*F*(1,57) = 5.9, *p* = 0.018) and for Group (*F*(1,57) = 13.8, *p* = 0.000) in ITT analyses. There was also a significant interaction effect of Time × Group (*F*(1,57) = 19.0, *p* = 0.000), which indicated a positive effect of the training on scores on exercise B. PP analyses showed similar results.

## Discussion

### Key findings

The results of this study show that participation in the training program improves the ability of OHPs to identify cognitions and perceptions. Participation also improves their ability to recommend evidence-based interventions toward workers to increase work participation.

The training had positive effects on the abilities of OHPs to involve cognitions and perceptions during their practice. According to Berkhof et al. (2011), Smith (2000) and Ataei et al. (2020), various components of the training were perceived as effective training strategies for teaching physicians. For example, the relevance of cognitions and perceptions and the training itself was emphasized, OHPs practiced identifying cognitions and perceptions in written cases, OHPs obtained feedback from the trainers on the exercises, and OHPs participated in group discussions. However, there were also characteristics of the training that might limit its effectiveness. For example, according to a review by Berkhof et al. (2011), most effective training programs last at least a whole day, while this training lasts only 4.5 h. Berkhof et al. (2011) also identified role-play as an important strategy for teaching physicians communication skills, which was not included in this training. Extending the training and including role-play exercises might increase the effects of the training.

To study the effects of the training, participants watched video vignettes of consultations between OPs and clients. Video vignettes of consultations with clients are commonly used for assessing or training the skills of physicians and physicians in training (Mazor et al. 2007; Baribeau et al. 2012; Bell et al. 2015; Arif et al. 2017). When developing our video vignettes, we followed the guidelines suggested by Hillen et al. (2013) in order to make realistic vignettes that would increase the external validity. Although the video vignettes resembled real-life consultations, the increased ability to identify cognitions and perceptions shown in the vignettes does not necessarily mean that identifying these person-related factors during real-life consultations will be easy. The clients in the video vignettes talked extensively about their problems and various cognitions and perceptions emerged, but previous research showed that clients’ disclosure is dependent on certain factors (De Wit et al. 2019a; Senteio and Yoon 2020; Greene 2009). Greene’s Disclosure Decision-Making Model shows that the decision to disclose information depends on, for example, the perceived risks of disclosing information, the relationship with the information receiver, and thoughts about how the receiver would respond to the information (Greene 2009). In addition, studies among workers with a chronic disease and primary care physicians showed that factors such as trust, listening, and asking open-ended questions are perceived as essential for disclosing information (De Wit et al. 2019a; Senteio and Yoon 2020). Thus, to identify cognitions and perceptions, knowledge about the various factors that can affect disclosure are essential for OHPs. During the training, OHPs learn about factors that can influence workers’ disclosure of cognitions and perceptions. However, the obtained knowledge concerning these factors was not studied in this trial. Therefore, it is possible that the effects of the training will be even more visible during real-life consultations, because OHPs who follow the training are better equipped to retrieve information from workers that is necessary to identify cognitions and perceptions.

### Strengths and limitations

A strength of this study is that the effect of the training was measured by means of a randomized trial in which the effects were tested using video vignettes that were based on real-life cases and therefore reflect true situations between physicians and clients. Another strength is that all home assignments were checked and scored by two researchers independently to increase the reliability of the scores.

However, there were also limitations. First, the number of participants in this study was relatively low, which could have influenced the power of this study. Besides, all participants were volunteers. These physicians might be more interested in involving cognitions and perceptions or better equipped to involve these factors during their practice than physicians in general. Another limitation is that because participants in the control condition had to complete two home assignments before the training—rather than one before and one after the training—it is possible that they were aware of the condition that they were in. However, we think that this did not influence their assignment scores. A final limitation is that overall, the scores on exercise B on recommending interventions were low; this especially concerns the pre-test and post-test scores of the control group. This indicates that it was hard for the physicians to think up interventions aimed at the cognitions and perceptions. Although these scores emphasize the need for the training program, more elaborate pilot testing of the home assignments could have prevented this floor effect.

### Implications for practice and future research

The training program for OHPs can help to identify the cognitions and perceptions of workers, which in turn can help OHPs to perceive when cognitions and perceptions are limiting work participation and to judge whether intervening on these factors is necessary. The training also increases OHPs’ ability to recommend evidence-based interventions. We are convinced that this can help them in their efforts to increase work participation. Because we only studied the effects of the training during simulated consultations between physicians and clients, additional studies should be conducted to test whether participation in the training program also affects the identification of cognitions and perceptions and recommendations in real-life consultations. In addition, it would be interesting to test whether the effects of the training are still apparent a couple of months after the training. It would be difficult to study the direct effect of the training program on work participation because there are many factors that can influence the work participation of workers with a chronic disease, such as the type of disease or disorder, the duration of the complaints, the type of work and the support from employers and colleagues. Nevertheless, further studies are needed to examine the effect of the training program on the work participation of workers with a chronic disease.

## Concluding remarks

The developed training program for OHPs increases their ability to identify the cognitions and perceptions of workers and to recommend evidence-based interventions. Participation in this training might help OHPs in their efforts to increase the work participation of workers with a chronic disease.

## Supplementary Information

Below is the link to the electronic supplementary material.

Supplementary file1 (DOCX 72 KB)

Supplementary file2 (DOC 219 KB)

## Data Availability

Data are available upon request.
