# Supplementary material for: Effects of a training program for occupational health professionals on the cognitions and perceptions of workers: a randomized controlled trial
Source: Int Arch Occup Environ Health. 2022 Jan 13;95(5):1059–66. doi: 10.1007/s00420-021-01823-7 (PMC9203404; doi:10.1007/s00420-021-01823-7)
Supplement: Supplementary file 1 — Supplementary file1 (DOCX 72 KB) [file 420_2021_1823_MOESM1_ESM.docx]

**Effects of a training program for occupational health professionals on the cognitions and perceptions of workers: a randomized controlled trial**

M. de Wit, PhD, B. Horreh, MSc, C. T. J. Hulshof, MD, PhD, H. Wind, MD, PhD, A.G.E.M. de Boer, PhD

Amsterdam UMC, University of Amsterdam, Department of Public and Occupational Health, Coronel Institute of Occupational Health, Amsterdam Public Health research institute, Amsterdam, The Netherlands

Correspondence Author: Mariska de Wit, Amsterdam UMC, Department of Public and Occupational Health, Coronel Institute of Occupational Health, PO Box 22700, 1100 DE Amsterdam, The Netherlands; +31(0)20-5665341; m.e.dewit@amsterdamumc.nl

**Online Resource 1.** Study flowchart

**Enrollment**

Assessed for eligibility (n = 92)

Excluded (n = 30)

- Not available on training data
   (n = 5)
- Not enough time (n = 3)
- No response (n = 22)

Randomized (n = 62)

Allocated to waiting-list control
(n = 30)

Allocated to training

(n = 32)

**Allocation**

**T0 Week 1**

Completed assignment T0 (n = 30)

Completed assignment T0 (n = 32)

Study drop-out (n = 3)

- Not enough time
  (n = 2)
- No reason provided (n = 1)

Allocated to training

(n = 29)

- Received training (n = 28)
- Received no training due to
  illness (n = 1)

Allocated to waiting-list control
(n = 30)

- Received no training (n = 29)
- Received training due to miscommunication (n = 1)

**Week 4**

**T1 Week 6**

Completed assignment T1 (n = 30)

Completed assignment T1 (n = 29)

Intention-to-treat (n = 29)
Per-protocol (n = 28)

Intention-to-treat (n = 30)
Per-protocol (n = 29)

**Analysis**

**Figure 1.** Study flowchart
